# Simultaneous totally robotic rectal resection and partial nephrectomy: case report and review of literature

**DOI:** 10.1186/s12957-020-01864-1

**Published:** 2020-05-04

**Authors:** G. Cochetti, A. Tiezzi, A. Spizzirri, D. Giuliani, J. A. Rossi de Vermandois, G. Maiolino, M. Coccetta, V. Napolitano, F. Pennetti Pennella, S. Francesconi, E. Mearini

**Affiliations:** 1 Department of Surgical and Biomedical Sciences, Urology Clinic Perugia–Terni, University of Perugia, Perugia, Italy; 2 Department of Surgical Specialties, SC General Surgery and Surgical Specialties, St Maria Hospital, Terni, Italy; 3 Department of Oncology, SC Pathological Anatomy, St Maria Hospital, Terni, Italy

**Keywords:** Synchronous tumors, Robotic, Colorectal tumor, Kidney tumor, Renal tumor, Colorectal cancer, Partial nephrectomy, Combined robotic surgery, Anterior rectal resection, Nephrectomy

## Abstract

**Introduction:**

The incidence of synchronous RCC and colorectal cancer is heterogeneous ranging from 0.03 to 4.85%. Instead, only one case of huge colon carcinoma and renal angiomyolipoma was reported. The treatment of synchronous kidney and colorectal neoplasm is, preferably, synchronous resection. Currently, laparoscopic approach has shown to be feasible and safe, and it has become the gold standard of synchronous resection due to advantages of minimally invasive surgery. We presented a case synchronous renal neoplasm and colorectal cancer undergone simultaneous totally robotic renal enucleation and rectal resection with primary intracorporeal anastomosis. As our knowledge, this is the first case in literature of simultaneous robotic surgery for renal and colorectal tumor.

**Case presentation:**

A 53-year-old woman was affected by recto-sigmoid junction cancer and a solid 5 cm left renal mass. We performed a simultaneous robotic low anterior rectal resection and renal enucleation. Total operative time was 260 min with robotic time of 220 min; estimated blood loss was 150 ml; time to flatus was 72 h, and oral diet was administered 4 days after surgery. The patient was discharged on the eighth post-operative day without peri- and post-operative complication. The definitive histological examination showed a neuroendocrine tumor pT2N1 G2, with negative circumferential and distal resection margins. Renal tumor was angiomyolipoma. At 23 months follow-up, the patient is recurrence free.

**Discussion and conclusion:**

As our knowledge, we described the first case in literature of simultaneous robotic anterior rectal resection and partial nephrectomy for treatment of colorectal tumor and renal mass. Robotic rectal resection with intracorporeal anastomosis surgery seems to be feasible and safe even when it is associated with simultaneous partial nephrectomy. Many features of robotic technology could be useful in combined surgery. This strategy is recommended only when patients’ medical conditions allow for longer anesthesia exposure. The advantages are to avoid a delay treatment of second tumor, to reduce the time to start the post-operative adjuvant chemotherapy, to avoid a second anesthetic procedure, and to reduce the patient discomfort. However, further studies are needed to evaluate robotic approach as standard surgical strategy for simultaneous treatment of colorectal and renal neoplasm.

## Introduction

Synchronous renal mass and colorectal cancer has been well described in the literature. Renal cell carcinoma (RCC) is associated with other primary malignancies in 16.1% [1]. Patients undergone surgery for colorectal cancer have a synchronous multiple cancer in 5.0% [2]_._ The incidence of synchronous RCC and colorectal cancer is heterogeneous ranging from 0.03 to 4.85% [3–5]. Moreover, patients with a history of colorectal cancer have a higher risk to develop RCC compared to the risk to develop colorectal cancer in patients with history of RCC (2.29 vs 1.14 standard incidence ratio) [6]. This association can be explained by the same environmental risk factors and the screening bias. The screening bias results from the frequent use of imaging such as computerized tomography (CT), magnetic resonance, and positron emission tomography, during the work-up of other malignancies that increased incidental diagnosis of asymptomatic synchronous urologic neoplasm [7]. A third explanation of this connection could be the sharing of the same genetic predisposition such as mismatch repair defect excluding the well-defined Lynch syndrome because in most of cases the criteria of Lynch syndrome are not met. Instead, the association between colorectal cancer and others renal mass is poorly reported: two cases of synchronous colorectal adenocarcinoma and renal oncocytoma have been reported [8, 9]; Kim et al. described a synchronous colorectal cancer and renal leiomyoma in a case series [10]. Only one case of huge colon carcinoma and right renal angiomyolipoma was reported [11]. Angiomyolipomas (AMLs) are the most frequent benign renal neoplasm, and they occur as sporadic in 80% of cases, but in the remaining 20% can occur in association with tuberosclerosis complex (TSC) or pulmonary lymphangioleiomyomatosis (LAM).

Usually, imaging is able to differentiate renal AMLs from carcinomas by ultrasound scan and/or CT scan due to the fat component. However, a minority of AMLs, named AMLs fat poorly, lack visually detectable fat on imaging, making it harder to distinguish from RCC [12].

The treatment of synchronous kidney and colorectal neoplasm is, preferably, synchronous resection. The open surgery was the best choice in the past years, but it was affected by two issues: first, wide surgical wound and the relative complications; second, high rate of peri- and post-operative morbidity [13]. Currently, laparoscopic approach has shown to be feasible and safe, and it has become the gold standard of synchronous resection due to advantages of minimally invasive surgery. We presented a case of 53-year-old woman affected by synchronous left renal neoplasm and colorectal cancer undergone simultaneous totally robotic renal enucleation and rectal resection with primary intracorporeal anastomosis. As our knowledge, this is the first case in literature of simultaneous robotic surgery for renal and colorectal tumor.

## Case presentation

The study was approved by the Ethics Committee of the University of Perugia, and a written informed consent was obtained by patient.

**Fig. 1:**
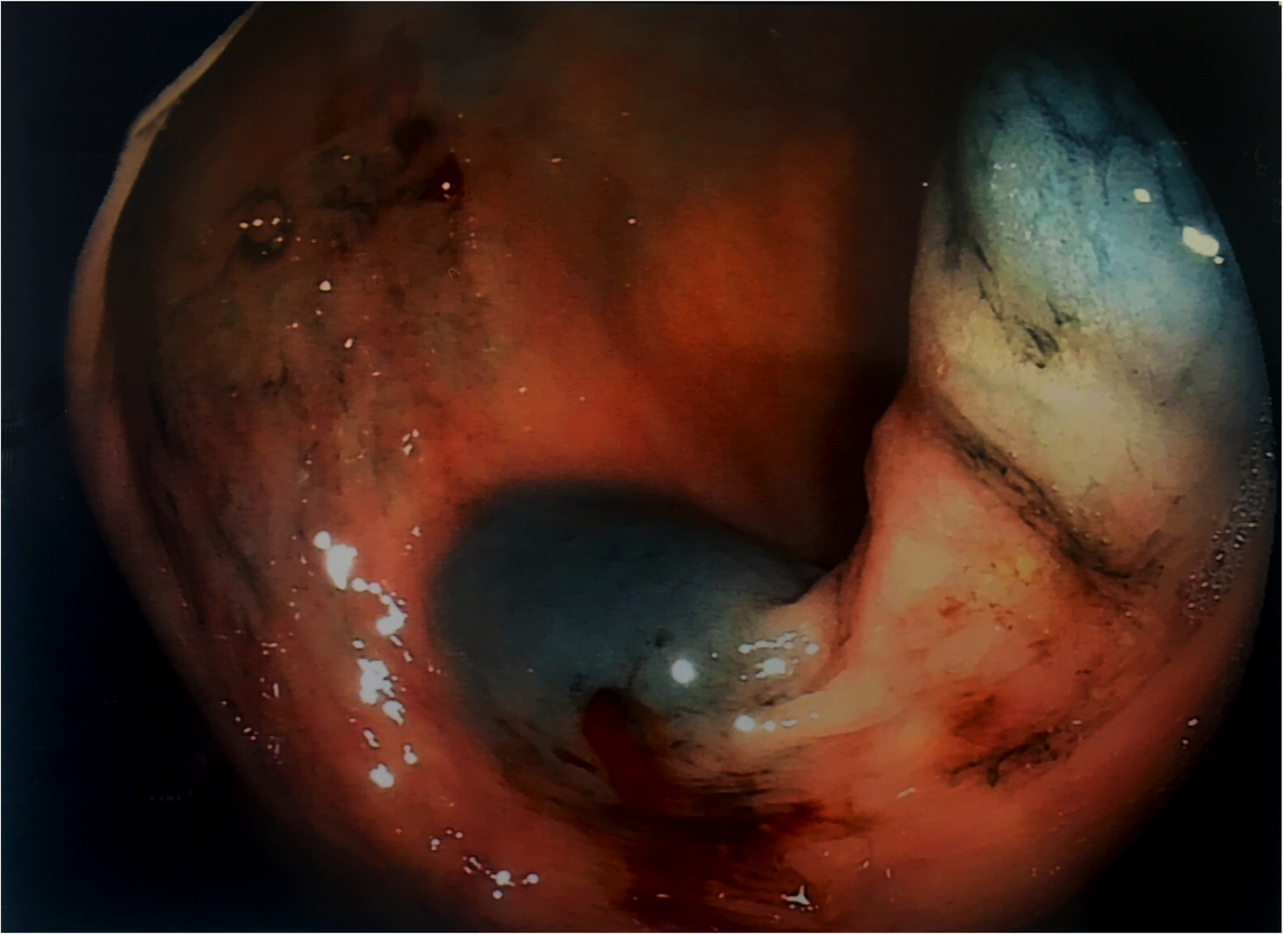
Colonoscopy view of rectal mass. Colonoscopy view showing a polypoid mass of 15 mm with bleeding surface

**Fig. 2:**
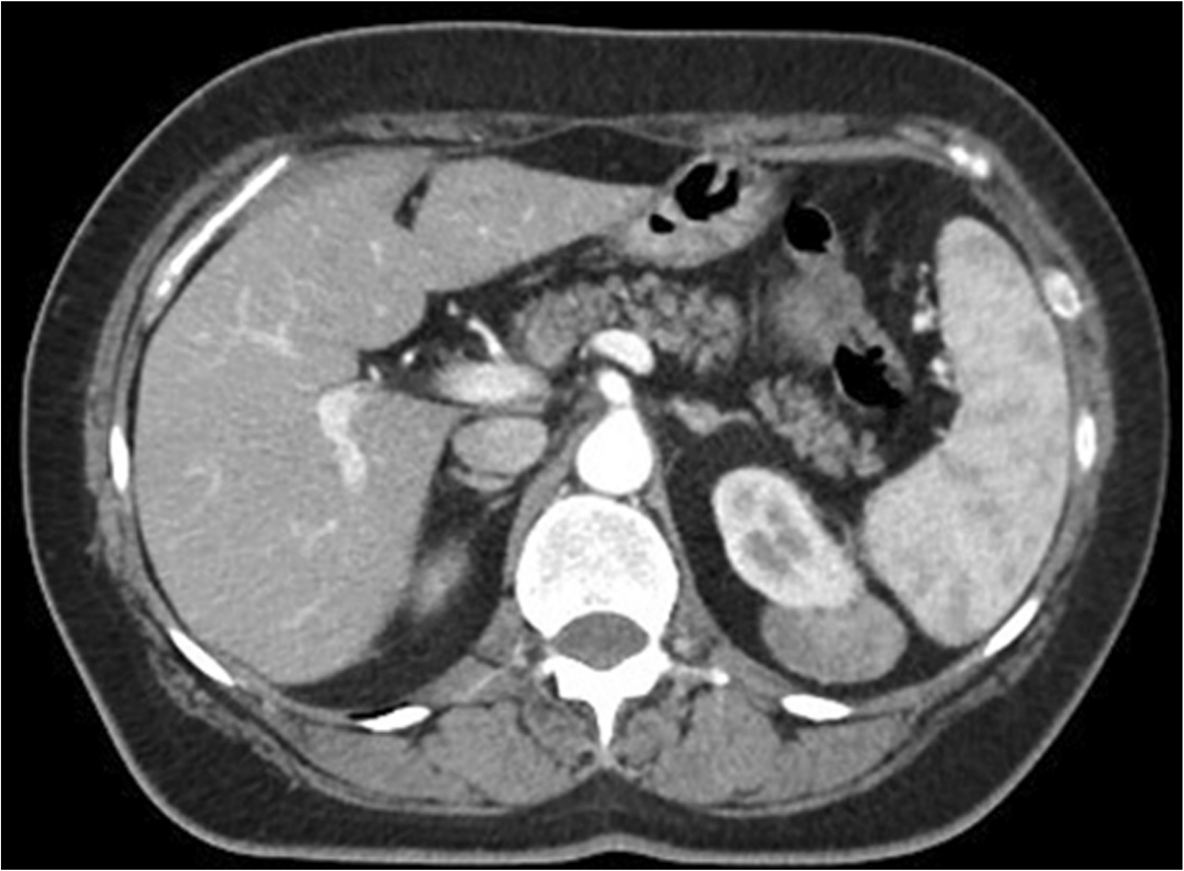
Computed tomography view of renal mass. Computed tomography (CT) scan shows a solid 5 cm mass in the left kidney with exophytic pattern placed in the upper pole of the kidney. The imaging features were consistent with clear RCC

**Fig. 3:**
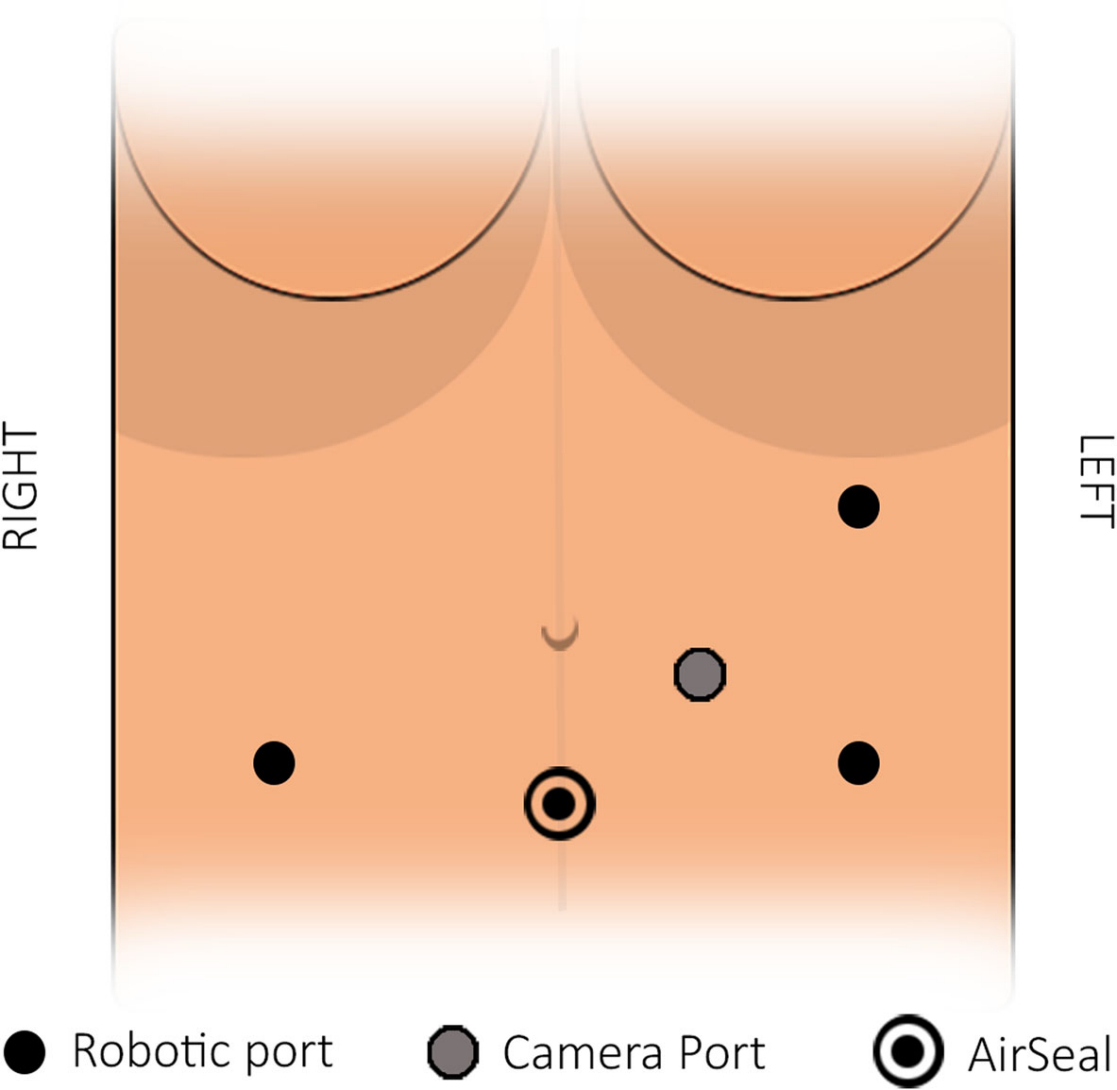
Port arrangement on anterior abdominal wall. A paraumbilical camera port (12 mm) was inserted. Three robotic ports (8 mm) were inserted at the left hypochondriac, in the left iliac fossa, and in the right iliac fossa. AirSeal System® trocar (12 mm) was placed in hypogastric region

**Fig. 4:**
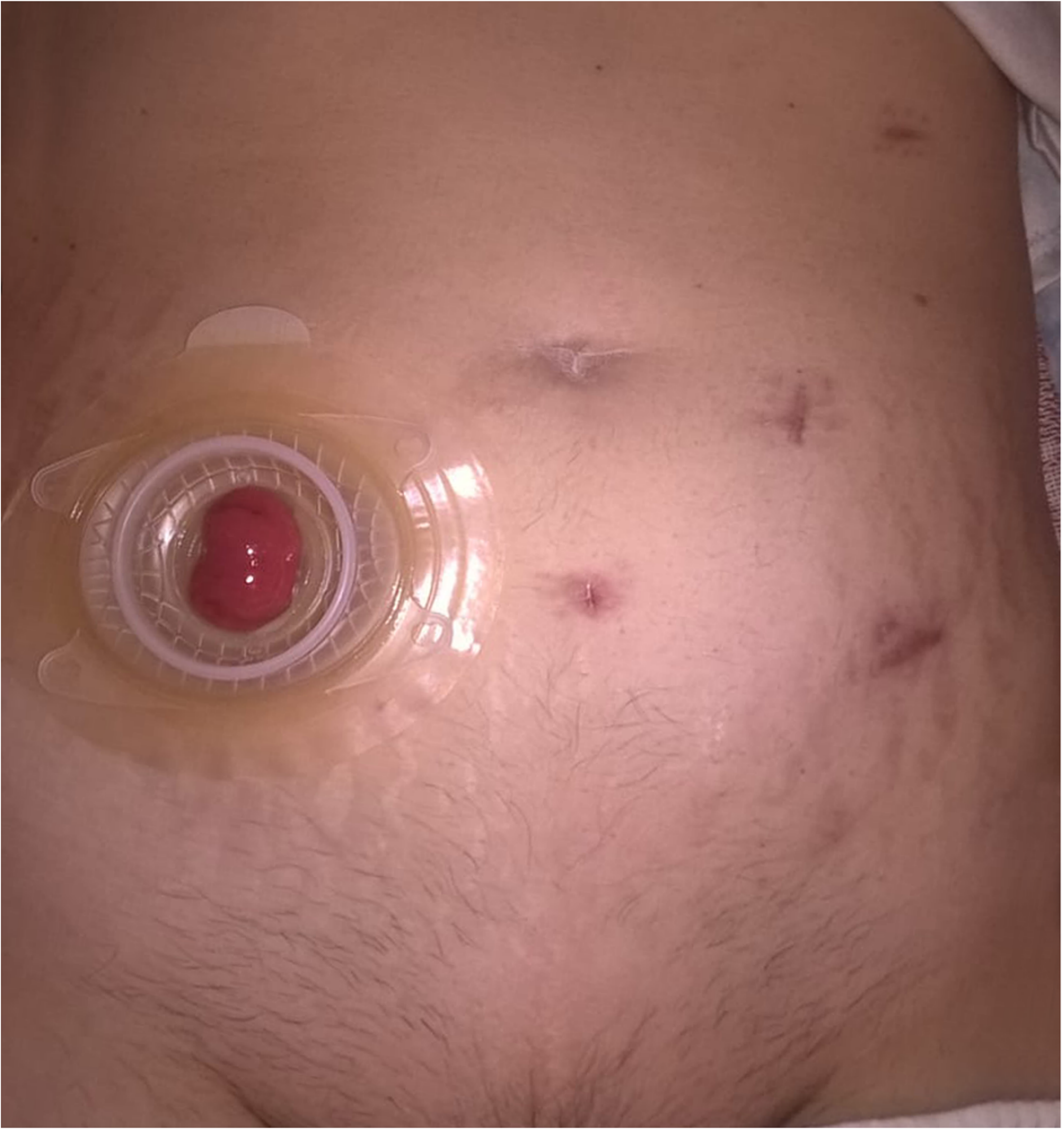
Abdominal wall port-site scars and ileostomy. Abdominal wall port-site scars and ileostomy in the right iliac fossa at 1 month after surgery

A 53-year-old woman was affected by colorectal cancer that was diagnosed through positive fecal occult blood test and colonoscopy with biopsy. The latter showed a polypoid mass of 15 mm with bleeding surface located in the recto-sigmoid junction (Fig. 1). Histological examination showed a neuroendocrine neoplasm with positive immunohistochemical staining for synaptophysin, chromogranin (Cg), CD56, cytokeratin AE1/AE3, and Ki67-labelling index of 4–5%. No comorbidity was reported. Physical examination was negative for colorectal or renal disease. Biochemical test, including renal and liver function, and urine analysis were normal too. Staging chest and abdominal CT scan did not show any other lesions except a solid 5 cm mass in the left kidney. The mass appeared exophytic in the upper pole and showed heterogeneous enhancing: the imaging features were consistent with clear RCC (Fig. 2). The patient underwent simultaneous robot-assisted rectal resection and renal enucleation using robot da Vinci® Xi (Intuitive Surgical Inc). Under general anesthesia, the patient was first placed in the right lateral decubitus position. A paraumbilical camera port was inserted. Three robotic ports were inserted at the left hypochondriac region along the hemiclavicular line, in the left iliac fossa about 2 cm medially to antero superior iliac spine (ASIS), and in the right iliac fossa about 2 cm medially to ASIS. AirSeal System® trocar was placed in hypogastric region (Fig. 3). Renal enucleation was performed without renal ischemia, after isolating the renal artery anyway according our previously published technique: renal artery was isolated, and a vessel loop was passed twice around it and pulled out extracorporeally parallel to the assistant trocar. On demand, the vessel loop could be tightened to obtain a progressive occlusion of the arterial lumen and, consequently, a renal hypoperfusion [14]. For this procedure, we placed the monopolar scissor in the left ASIS trocar, the bipolar forceps in the left hypochondriac region trocar, and the prograsp in the right ASIS trocar used for medialization of the left colon. For low anterior rectal resection, the patient was placed in lithotomy position, and we placed the monopolar scissor in the right ASIS trocar, bipolar forceps in the left ASIS trocar, and prograsp in the left hypochondriac region trocar. The rectal resection was carried out up to lower rectal segment through total meso-rectal excision and nerve sparing technique. The end-to-end anastomosis was carried out according to Knight Griffen technique, and a loop ileostomy was performed in site of the trocar in the right iliac fossa (Fig. 4). The specimens were retrieved in an endo-bag through a transverse colpotomy that was closed by robotic intracorporeal suture.

## Results

**Fig. 5:**
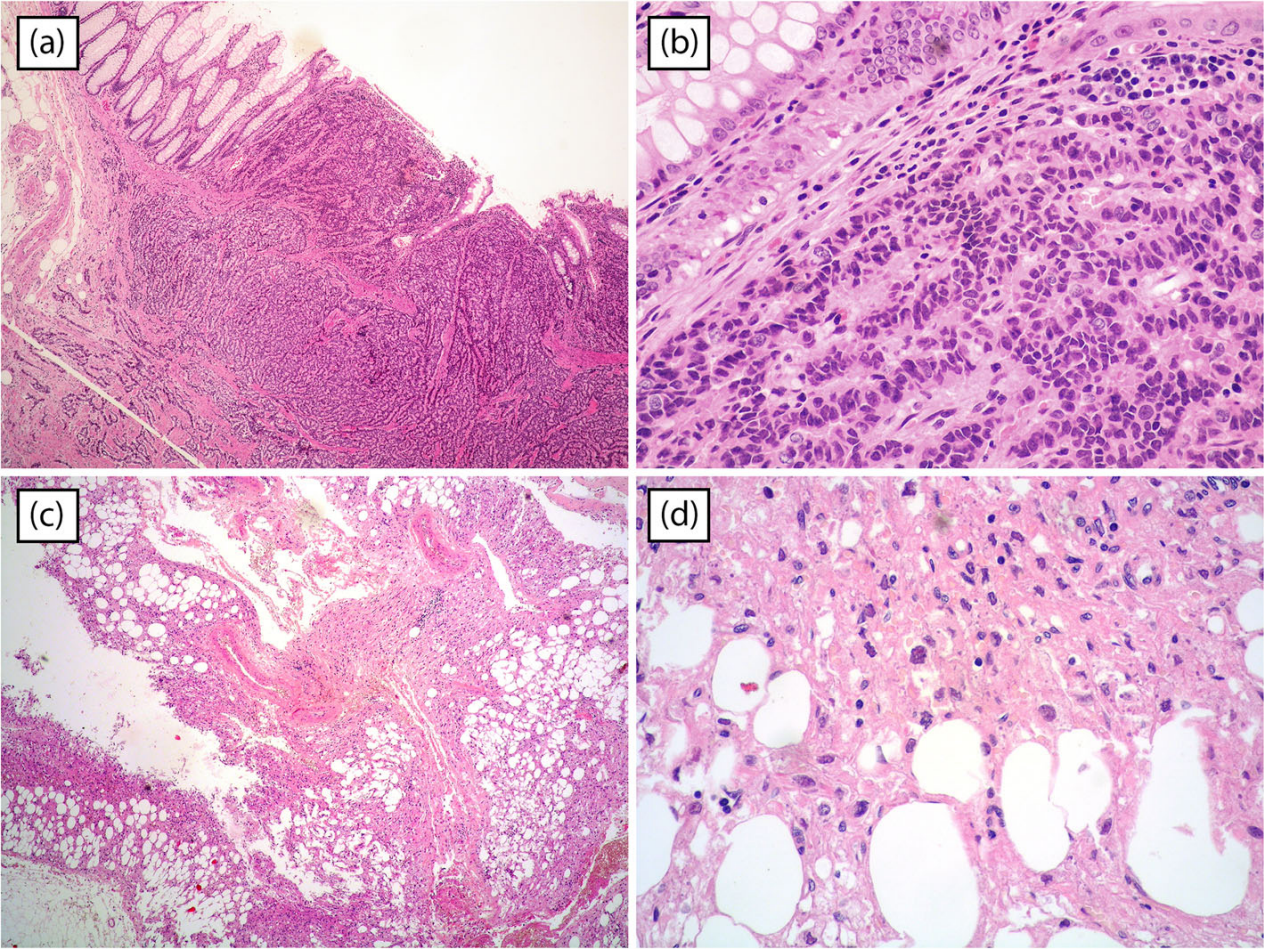
Histopathology specimen. Neuroendocrine tumour in hematoxylin and eosin (HE) stain with original magnification × 40 (**a**) and × 400 (**b**). Angiomyolipoma in HE stain with original magnification × 40 (**c**) and × 400 (**d**)

Total operative time was 260 min with robotic time of 220 min; estimated blood loss was 150 ml; time to flatus was 72 h, and oral diet was administered 4 days after surgery. The patient was discharged on the eighth post-operative day without peri- and post-operative complication. The definitive histological examination showed a neuroendocrine tumor pT2N1, G2, with negative circumferential and distal resection margins. Renal tumor was angiomyolipoma, and the immunohistochemistry was positive for anti-MART-1 and HMB-45, anti-actin, and alfa-smooth, but negative to calponin, desmin, S-100, and cytokeratin8/18 (Fig. 5). CD31 was positive in vessels, and Ki67/Mib-1 was < 1%. At 3 months after surgery, the bowel integrity was restored. At 23 months follow-up, the patient is recurrence free.

## Discussion and conclusion

Synchronous colorectal and renal tumor is not frequent. Environmental and genetic factors and the screening bias are the reasons behind this association. Some authors recommend use of ultrasound examination, CT, and magnetic resonance imaging to exclude synchronous asymptomatic renal lesions in patients with colorectal cancer to improve patients’ survival rates [5]. Such as others synchronous tumors, there are not approaches based on prospective trial evidence, and they are therapeutic dilemmas. Synchronous multiple primary tumors should be discussed in multidisciplinary team, and the patient should be informed about therapeutic challenges [15]. Many reports on synchronous tumors suggest that treatment should be performed simultaneously, especially if the lesions are relatively small and may be treated by unique access. This strategy is recommended only when patients’ medical conditions allow for longer anesthesia exposure. Otherwise, first treatment should be directed to the more aggressive lesion. The advantage to treat two neoplasms at the same time is to avoid a delay treatment of second tumor, especially in younger patients [16]_._ Finally, a combined surgery reduces the time to start the post-operative adjuvant chemotherapy that is longer if two different procedures are performed separately [17]. Moreover, a second anesthetic procedure may be avoided, and discomfort of patients could be reduced [18]. Open surgery was the gold standard in the past years. Cullinane et al. reported seven cases of simultaneous colorectal and renal carcinoma treated by open combined surgery without significant morbidity [9]. Somani et al. suggested that post-operative morbidity after combined surgical procedures for RCC and other intra-abdominal pathologies in comparison with surgical procedures for RCC alone is slightly higher but not was statistically significant [19]_._ For synchronous renal and colorectal tumors, the laparoscopic approach is now a well-established treatment. Indeed, laparoscopy allows to perform simultaneous interventions by the same accesses and offers a magnified visualization, an early control of the organ’s peduncles, and a screening for disseminated disease [7]. Many single cases or small case series have been reported in literature for synchronous ipsilateral and controlateral colorectal and renal disease treated by laparoscopy (Table 1). Kim et al. reviewed data from 93 patients with colorectal cancer and undergone simultaneous multiple organ resection, and 1090 patients undergone laparoscopic right hemicolectomy or low/anterior resection for colorectal cancer. The authors compared the intra-operative and short term outcomes between two groups, and they showed longer operative time in the combined group (189.6 min vs 166.9 min, *P* value 0.048 for patients underwent a right hemicolectomy, 178 min vs 228.1 min, *P* value ≤ 0.001 for patients underwent a low/anterior resection), while intra-operative outcomes such as the intra-operative complications, open conversion rate, and post-operative morbidity were similar [10]. The authors concluded that simultaneous laparoscopic surgery for synchronous abdominal lesions is safe and feasible in colorectal cancer patients; they demonstrated that the combined surgery allowed to decrease the length of stay and overall morbidity. Combined kidney and colorectal surgery provides an early post-operative recovery and decreased morbidity when associated with laparoscopic approach. Moreover, combined procedures in laparoscopic surgery consent to reduce the risk of pulmonary and cardiac burden related to a single anesthesia instead of two, to obtain an earlier return to work activities and better cosmetic result, in addition to a better cost-effective due to more efficient use of hospital beds [10, 27]. Simon et al. reported their experience with 5 cases of synchronous laparoscopic resection of colorectal cancer and renal or adrenal mass. The authors highlighted that this combined laparoscopic surgery was safe and feasible, with favorable peri-operative and oncologic outcomes: they reported median operative time of 420 min, median blood loss of 1000 ml, median number of blood transfusion of 1.5 unit, median removed lymph nodes of 21, no major complications, and median hospital stay of 11 days; no recurrences were observed [7].

**Table 1:** Colorectal and renal masses treated by laparoscopy reported in literature

Reference	Sex, age (year)	Colorectal neoplasm	Renal neoplasm	Simultaneous procedure		Blood loss (ml)	Operative time (min)
				First time	Second time		
Kim et al. [20]	M, 55	Mid-sigmoid ADC	Right clear cell RCC	LRN	Laparoscopic sigmoidectomy	100	355
Ng et al. [13]	M, 80	Descending-sigmoid colon junction ADC	Left chromophobe RCC	Laparoscopic left hemicolectomy	LLN	1500 (mostly from the left renal vein)	370
Napolitano et al. [21]	M, 74	Left colonic ADC	Left RCC	LRN	Laparoscopic left hemicolectomy	300	270
Ng et al. [7]	M, 73	Sigmoid carcinoma	Right RCC	Laparoscopic sigmoid colectomy	LRN	200	420
	M, 80	Descending colon carcinoma	Left RCC	Laparoscopic left hemicolectomy	LLN	1500	370
Nishiyama et al. [22]	M, 65	Descending colon ADC	Left ureteral grade 2 transitional cell carcinoma	Laparoscopic left nephroureterectomy	Laparoscopic descending colectomy	158	442
Veenstra et al. [23]	F, 70	Ascending colon ADC	Right clear cell RCC	LLN	Laparoscopic right hemicolectomy	100	210
Campanati et al. [24]	M, 68	Descending colon ADC	Right Clear Cell RCC	LRN	Laparoscopic left hemicolectomy	100	450
	M, 70	Sigmoid ADC	Left Clear Cell RCC	LLN	Laparoscopic left hemicolectomy	150	380
Fazzin et al. [25]	F, 79	Sigmoid ADC and right colon tubulous-villous adenomas	Right RCC	Laparoscopic right hemicolectomy and sigmoidectomy	LRN	N.A.	N.A.
Takahashi et al. [26]	F, 70	Ascending colon ADC	Right RCC	Laparoscopic right hemicolectomy	LRN	60	450
O’Sullivan et al. [27]	M, 73	Mid-transverse colon ADC	Left clear cell RCC with minor component of solid-variant papillary	LLN	Laparoscopic complete mesocolic excision	N.A.	N.A.
Martin Arnau et al. [18]	M, 63	Sigmoid ADC	Left Type 2 papillary RCC	Laparoscopic sigmoidectomy	LLN	0	300
	M, 67	Sigmoid ADC + colon polyposis	Right clear cell RCC + left cystic RCC	Subtotal colectomy	Laparoscopic heminephrectomy (partial left nephrectomy by retroperitoneoscopy was performed few days before)	900	420
	M, 71	Rectal ADC	Left type 2 papillary RCC	Lower anterior resection	LLN	N.A.	N.A.
	M, 62	Right colon ADC	Transitional cell carcinoma of the ureter	Laparoscopic right nephroureterectomy	Laparoscopic extended right hemicolectomy	300	360
Lee et al. [28]	F, 71	Ascending colon ADC	Left non-functioning kidney with hydronephrosis	Laparoscopic right hemicolectomy	LLN	300	275
	M, 77	Descending colon ADC	Left clear cell RCC	Laparoscopic left hemicolectomy	LLN	250	395
Imagami et al. [17]	M, 77	Transverse colon ADC	Left renal cell carcinoma of the left kidney	Robotic-assisted partial nephrectomy	Laparoscopic transverse colectomy	50	510
Tokuda et al. [29]	M, 83	Cecal ADC	Left renal clear cell carcinoma	Laparoscopy ileocecal resection	LLN	30	560
This case	F, 53	Recto-sigmoid junction neuroendocrine tumor	Left renal angiomyolipoma	Robotic-assisted partial left nephrectomy	Robotic-assisted low anterior rectal resection	150	260

*LRN* laparoscopic right nephrectomy, *LLN* laparascopic left nephrectomy, *ADC* adenocarcinoma, *RCC* renal cell carcinoma

The robotic approach to synchronous renal and colorectal is a new challenge and, as our knowledge, our case is the first case in literature of simultaneous robotic anterior rectal resection and partial nephrectomy for treatment of renal and colorectal tumor.

In our case, for kidney tumor treatment, we performed a robotic enucleation without renal ischemia in order to obtain maximum preservation of healthy renal tissue and, consequently, of renal function. Indeed, the deterioration of kidney function after partial nephrectomy is due to both the loss of healthy parenchyma and the damage related to ischemia. The renal enucleation is an oncologically safe and effective technique that permits to remove the tumor using the avascular cleavage plan due to the fibrous pseudocapsular reactive tissue surrounding the tumor: in this way, a maximum sparing of healthy tissue may be achieved. Moreover, we preferred not to clamp the renal artery during enucleation to avoid the damage due to ischemia, since kidney function is already affected by prolonged anesthesia of the combined surgery. However, a vessel loop was passed twice around the renal artery and pulled out extracorporeally in order to apply a progressive renal hypotension on demand. Indeed, in case of significant bleeding, the arterial flow to the kidney can be reduced by assistant pulling vessel loop: this preserve the safety of the intervention even in combined surgery [14, 30].

Boni et al. showed the feasibility and safety of combined pancreatic metastasis and partial nephrectomy in one single robotic combined resection without replacement of the trocars [31]. In literature, only one case series was reported about combined laparoscopic and robotic surgery for synchronous colorectal and genitourinary cancer. Imagami et al. retrospectively analyzed the surgical outcomes from a series of 3 cases of laparoscopic colorectal resection combined with robotic partial nephrectomy (n 1) or radical prostatectomy (n 2). They stated that the minimally invasive combined surgery was safe and feasible and allowed prompt initiation of adjuvant chemotherapy [17]. In our case, we used five ports confirming that for synchronous colorectal and urogenital tumor the sharing ports are possible also by robotic approach. Robotic technique allows to maintain the advantages of laparoscopy and to obtain more benefits as magnification of the operative field through three-dimensional vision and high definition, more accurate movements by EndoWrist® instruments (Intuitive Surgical Inc) with 7° of motion, primary surgeon camera control, and elimination of the tremor. Thereby, this technology consents to reproduce the same surgical steps of traditional surgery with the benefits of a minimally invasive technique, overcoming the limitations of the laparoscopy: an unstable video camera, limited range of instruments’ movements, two-dimensional imaging, and poor ergonomics for the surgeon [32, 33]. The robotic system facilitates the identification of anatomical structures and makes easier some complex surgical step in a narrow space such as the pelvis. These advantages could facilitate complex combined surgery for synchronous tumor.

As our knowledge, we described the first case in literature of simultaneous robotic anterior rectal resection and partial nephrectomy for treatment of colorectal tumor and renal mass. Robotic rectal resection with intracorporeal anastomosis surgery seems to be feasible and safe even when it is associated with simultaneous partial nephrectomy. Many features of robotic technology could be useful in combined surgery. However, further studies are needed to evaluate robotic approach as standard surgical strategy for simultaneous treatment of colorectal and renal neoplasm.

## Data Availability

The authors declare that all data supporting the findings of this study are available within the article.

## Abbreviations

RCC: Renal cell carcinoma
CT: Computerized tomography
AMLs: Angiomyolipomas
TSC: Tuberosclerosis complex
LAM: Lymphangioleiomyomatosis
Cg: Chromogranin
ASIS: Anterior superior iliac spine
